# Proactive personality and organizational support in television industry: Their roles in creativity

**DOI:** 10.1371/journal.pone.0280003

**Published:** 2023-01-10

**Authors:** Syamsul Hidayat, Zukhruf Febrianto, Anis Eliyana, Unggul Purwohedi, Rachmawati Dewi Anggraini, Alvin Permana Emur, Marziah Zahar

**Affiliations:** 1 Universitas Negeri Jakarta, East Jakarta, DKI Jakarta, Indonesia; 2 Department of Management, Universitas Airlangga, Surabaya, East Java, Indonesia; 3 Research and Publication, PT Usaha Mulia Digital Indonesia (PT UMDI), South Jakarta, DKI Jakarta, Indonesia; 4 Department of Management, Universitas Indonesia, Depok, West Java, Indonesia; 5 School of Business Management, Universiti Utara Malaysia, Sintok, Kedah, Malaysia; The Islamia University of Bahawalpur Pakistan, PAKISTAN

## Abstract

Employee creativity is important for TV companies because it can improve organizational performance and increase success and survival based on the ability to create innovations. In response to this, field reporters who work for TV companies also need high creativity in facing the challenges of their work and in creating innovations. This research aims to test the roles of perceived organizational support, proactive personality, the meaning of work, and work engagement in affecting employee creativity. The total numbers of respondents were 119 selected from 14 private television companies in Indonesia. The method in this study is a quantitative approach using the Partial Least Square (PLS) analysis tool with the SmartPls 3.0 application. Most of the hypotheses of this study show significant results. However, there is one finding that a proactive personality is not able to strengthen employee creativity. This indicates that employees’ behavior at work is sometimes different from the personality. This study is the first to look at news reporters’ employee creativity using the suggested model. As a result, organizations can use the study’s findings as a starting point to determine the best strategy for fostering creativity within their workforce.

## 1 Introduction

Seeing the need for information in society, television still becomes a high-demand mass media. Though television still survive amid the emergence of new mass media platform recently, but the increasing number of new platforms such as Youtube and Netflix have raised challenges. As a result, television channels as part of business must maintain and improve their ratings to survive and generate profits. One well-known rating system is Nielsen Ratings. It is a system for television channels developed by Arthur C. Nielsen, a market analyst through his organization, Nielsen Media Research. A high Nielsen rating represents more viewers and eventually advertisements. Therefore, this has an impact on the profits generated by the television channel.

Television still maintains a high level of viewing and is anticipated to continue for at least the next few decades, despite the claim that it is a conventional media that has been disrupted by online media [1]. Even Wolff acknowledges that some aspects of television media, such as authenticity and reliability, cannot be replicated by digital media in his book "Television is the New Television: The Unexpected Triumph of Old Media in the Digital Age" [2]. Furthermore, television that already exists has a chance to compete with digital media, which is thought to just digitalize what already exists and has not been able to develop the characteristics of broadcasting and content development. Additionally, user-generated content on digital platforms has a lower quality of information and tends to be more amateurish than professional news reporting on television. Additionally, television still becomes the primary option for viewers who reside in distant places without access to the internet as an entertainment and information source. The internet, as is well known, needs a network from an operator with a currently constrained service area. In the meantime, 20% of Indonesia’s population is still without internet access.

Television channels are ranging from government and private-owned, national and international channels, but practically all of them provide news programming, as news is a daily necessity that people cannot live without. People increasingly monitor both national and international television stations for news as a result, television stations must adapt their programming in light of globalization. The news reporters are undoubtedly impacted by this, who must be alert and perceptive when choosing worldwide phenomena that worth broadcast.

Employee creativity may be influenced by perceived organizational support, a proactive personality, and meaning of work [3]. Previously, a link between work engagement and perceived organizational support was also proved [4]. According to a former study, proactive personalities and work engagement go hand in hand [5]. Likewise, as asserted in other research there was a connection between employee creativity and work engagement [6]. Thus, this study attempts to elaborate those studies by taking into account five variables, including perceived organizational support, proactive personality, meaning of work, work engagement, and employee creativity, that have been empirically correlated in other studies.

The employee’s view of the organization is considered important in determining performance at work. Perceived organizational support is defined as a general belief that the organization cares about the contributions and welfare of its employees, thus making employees feel safe and comfortable in the organization [7]. Thus, this study will discuss further perceived organizational support and its effect on field news reporters. Then, another assumption arises those proactive individuals have better work performance than those who are not proactive. Furthermore [8], suggests that a proactive personality refers to someone who is always looking for opportunities, shows initiative, takes action when needed, and maintains an attitude until the desired change is achieved. In his work titled "Man’s Search for Meaning," Viktor Emil Frankl asserts that meaning plays a central role in the lives of an individual. Similarly, in the workplace, the meaning of work will undoubtedly influence work performance. Meaning of work can determine how employees interpret their work and organization, how they do their duties, and what they experience within the organization [9]. Furthermore, research conducted by [10] suggests work engagement as an employee’s attachment to their work, which motivates them to exert more effort since they view the work as an extension of themselves.

Due to the significance of creativity in the entertainment and content creation industries, especially television programming, it is crucial to undertake a study examining the elements that drive creativity. Creativity is a crucial quality for a field reporter to possess. This is due to the fact that field reporters are frequently confronted with unforeseen circumstances, such as sudden changes in coverage plans, which demand reporters to utilize their inventiveness. In addition, when conducting research on coverage material, reporters must be able to create news targets that are relevant and engaging in order to raise ratings while preserving aspects of authenticity and truth. Several field reporters, including television reporters, indicated that they were responsible for researching coverage material, conducting field research, arranging coverage treatment, carrying out production, and making rough cuts or rough editing. This demonstrates that a field reporter must be adaptable and creative in order to endure shifting field conditions.

Despite the critical role that television news reporters play in the news reporting process, there are unfortunately still very few studies that concentrate on them. Since television news reporters must communicate the news in auditory and visual formats that viewers can directly perceive, which raises the need for creativity on their part, the role of a news reporter comprises challenging obligations and responsibilities. Therefore, it is vital to comprehend the practical factors that influence television news reporters’ creativity.

This study aimed to perform additional research on television news reporters in light of the numerous earlier studies that have focused on creativity. As previously mentioned, other researches have sought to identify variables that may significantly influence employee creativity, but this study is the first to use the model proposed here. It is expected that it will be either theoretically useful for studies involving related variables or practically useful for insights on how to stimulate employee creativity.

## 2 Literature review

### 2.1 Theoretical basis

#### 2.1.1. Perceived organizational support

The definition of perceived organizational support is an employee’s general acknowledgment of the extent to which the organization values the contribution and welfare of employees who are perceived to provide meaning and purpose to the employees’ lives [11]. According to [12] perceived organizational support is also known as the global trust that an employee has made regarding their assessment of organizational policies and procedures. Perceived organizational support demonstrates the psychological process that occurs when employees get knowledge about their social environment, based on organizational support theory and social exchange theory, which is regarded as the primary theoretical foundation [13]. According to [14] perceived organizational support is related with organizations’ awareness regarding the extent to which employees’ involvement is valued and how employees’ view their organization having a concern for their welfare. Therefore, employees with high perceived organizational support tend to build a better life purpose and meaning in their career It can then be emphasized that perceived organizational support is employees’ perception of organizational policies, the justice given to employees and how the organization pays attention to socio-emotional needs. Perceived organizational support is also a form of employees’ expectations after what they have given to the organization [3,15].

#### 2.1.2. Proactive personality

Proactive personality show individual behavior to encourage change, recognize opportunities, and control situations to take advantage of those opportunities [16]. Individuals with proactive personalities exhibit a personal disposition to engage in active role orientation, such as initiating change and manipulating their environment, which makes the employee promise change through continued action until significant changes in the achievement of their goals occur [16]. According to [17] that individuals with a proactive personality is a person who is relatively unconstrained by situational forces and capable of influencing environmental change. The proactive personality type can reflect the extent to which individuals tend to find opportunities to make changes in the workplace and act in bringing about these changes. Hence, proactive personality is dubbed as "an anticipatory action that employees use to influence themselves and/or their environment". It should be stressed that individuals with proactive personalities constantly seek change in order to further their aims. Additionally, proactive personality in this study is defined as a personality trait that prefers to take the initiative when confronted with particular circumstances or activities [18].

#### 2.1.3. Meaning of work

Meaning of work is defined as a balance or alignment between employee characteristics and employee expectations, which occurs when employees dedicate themselves to value and meaning at work [3]. The study also states that the meaning of work is a construction that depends on the characteristics of individuals and their experiences, as well as their perceptions of how work has a meaning. According to [9] meaning of work is a fundamental factor that determines how members interpret their work and organization, how they perform their duties, and what they experience in the organization. Meaning of work can contribute to an individual’s perception of being motivated, fulfilled, and committed in life in general [19]. Thus, the meaning of work can be expressed as an alignment between the beliefs, values, and behavior of employees, which occurs when the work target is following the values or standards of the employees themselves [20]. A person’s level of work meaning reveals how much they value the purpose and utility of their employment. As a result, this study considers meaning of work as a gauge of how much employees believe their work is significant, worthwhile, and important in their life [21].

#### 2.1.4. Work engagement

Work engagement has been considered as an important predictor of employee attitudes, performance, and behavior [22]. Also supported by [23] who stated that employee work engagement is important because it determines employee well-being and is associated with many motivating work-related outcomes. Work engagement is defined as a positive work-related state of mind that individuals perform related to work characterized by vigour, dedication, and absorption [24]. In the workplace, vigor increases energy levels and psychological toughness. Dedication is characterized by drive, enthusiasm, and challenge. Absorption is characterized by a person’s complete focus on one task and his refusal to interrupt it. [25]. Employees with work engagement are enthusiastic, energetic, and fully absorbed in their work and act to advance the goals and prestige of the organization [26]. It may be noted that previous research examined work engagement in depth, starting from the factors that underlie it to something that can be altered by it [27]. This study makes use of Schaufeli’s concept of work engagement, that is a productive state of mind manifested by people who exhibit energy, dedication, and absorption [27].

#### 2.1.5. Employee creativity

The notion of creativity refers to creative work that is produced and is considered a new work that is accepted to be a work that can be maintained or useful and satisfying by a group at one time [28]. Employee creativity is defined as the conceptualization, production, and development of new and useful ideas, as well as processes and procedures by employees or a group of people working together [29]. Creativity involves bringing something new to an organization, which may mean something unique, and unusual such as originality, thinking outside the box, new perspectives, and contributing something that didn’t exist before [30]. Employee creativity can find the hidden needs of others and handle problems creatively and effectively, which ultimately creates superior performance [31]. Thus, employee creativity is capable of conceptualizing, producing, and developing new and useful ideas, involving the ability and capacity of employees to create and develop new and useful thoughts about company products, services, and procedures [14]. The definitions include creative solutions to problems or creative business strategies and creative changes in business processes. Employee creativity is, in other words, the degree of creativity that employees possess in relation to the generation of ideas and ideas. Employee creativity, however, goes beyond just that. It also has to do with how well people use their skills and aptitudes to solve issues quickly and effectively and make decisions. Additionally, this study used Tierney’s definition of employee creativity, which takes into account each person’s assessment of their own level of originality [32]. The definition is thought to be the most acceptable because this study cites two components, intrinsic and extrinsic, that are connected to employee creativity. Engagement, the meaning of work, and a pro-active attitude are intrinsic variables, whereas external elements connected to organizational support.

### 2.2 Hypothesis development

#### 2.2.1 Perceived organizational support and employee creativity

Perceived organizational support can meet various socio-emotional needs, including affiliation, self-esteem, emotional support, and social approval which can be correlated with creativity and innovation, because perceived organizational support can affect employee creativity through intrinsic and synergistic extrinsic motivation [33]. According to the study, employees with perceived organizational support are likely to increase their sense of responsibility to achieve organizational goals which refer to a creativity that is formed from setting difficult goals, choosing challenging tasks, and persisting when obstacles are encountered. It is known that in a competitive business environment, organizations need to encourage the creative performance of employees to survive, and perceived organizational support plays an important role in employee creativity, as it tends to increase the likelihood of creative outcomes [14]. According to [34] perceived organizational support can increase employee creativity behavior by increasing employee interest in their work. By referring to components related to creativity and innovation which are dynamic, [33] have assumed that individuals who receive support from perceived organizational support will change their intrinsic and extrinsic motivation. Research conducted by [33] has also revealed that there is a positive correlation between perceived organizational support and employee creativity through studies conducted. This study needs to examine how perceived organizational support affects employee creativity, especially on news reporters at Indonesian private television stations because in previous studies, the subjects studied were mostly centered on the hospitality, banking, and even multisector sectors [14,29,33]. From the descriptions mentioned above, this study hypothesizes that:

H1: Perceived Organizational Support has a significant effect on Employee Creativity

#### 2.2.2 Proactive personality and employee creativity

Employees with a proactive personality can initiate changes within the institution to achieve the desired goals, one way to do this is by manipulating work arrangements to improve performance and stimulate innovation [16]. Research by [16] also argues that a workforce with a proactive personality can seek every opportunity to detect new methods, demonstrate their skills, and explore modern work techniques, therefore, employee creativity is the right result of a proactive personality. Individuals with a proactive personality do not passively adapt to all aspects of the environment in which they live but are motivated to find new and better solutions for various procedures or processes that they consider ineffective in improving their current situation [17]. Hence, individuals with proactive personalities have a real desire to actively shape the surrounding environment to better suit their needs, and proactive personalities are more likely to show creative behavior [17]. According to [16,17] proactive personality is significantly related to employee creativity. Research by [35] suggests that proactive personality affects employee creativity through informal leadership formed by individuals who have proactive behavior. Individuals with proactive personalities Those with stronger inner thoughts and feelings tend to adopt positive strategies when they engage in the problem-solving process and can generate new ideas, and as a result, generate further resource gains such as employee creativity [36]. From the descriptions mentioned above, this study hypothesizes that:

H2: Proactive Personality has a significant effect on Employee Creativity

#### 2.2.3 Perceived organizational support and meaning of work

Employee empowerment in the form of perceived organizational support can have a positive impact on the meaning of work. This is evidenced in research [3] which found a correlation between perceived organizational support and meaning of work where employees who received perceived organizational support felt that their work had more meaning and meaning. According to [9] that the more individuals feel the meaning of work, the higher the likelihood that their perceived organizational support will increase. This can happen because perceived organizational support shows employee perceptions related to the extent to which the organization recognizes and appreciates its efforts and values, and cares about its welfare in an organization. If employees feel that they are supported by their organization, they will contribute more to organizational outcomes as a way of responding to that organizational support, and they will feel empowered by the support, knowledge, resources, and opportunities such as formal and informal strengths provided by their organization. to be able to feel work becomes more meaningful [3]. From the descriptions mentioned above, this study hypothesizes that:

H3: Perceived Organizational Support has a significant effect on the Meaning of Work

#### 2.2.4 Perceived organizational support and work engagement

Perceived organizational support reflects a growing type of support that can generate a sense of obligation to care for the well-being of the organization and help the organization achieve its goals based on the norm of reciprocity [37]. The research shows that repeated good treatment received from the organization can increase the employee’s sense of obligation to help the organization achieve its goals. Therefore, it makes sense that employees who have perceived organizational support high, leading to work engagement that can contribute more to job performance, compared to those who have perceived organizational support low [37]. According to [23,37] perceived organizational support has a significant effect on work engagement. This is shown by the relationship when employees believe that their supervisors and organizations care about their well-being, they are more likely to fulfill their obligations by being more involved in the workplace [23]. From the descriptions mentioned above, this study hypothesizes that:

H4: Perceived Organizational Support has a significant effect on Work Engagement

#### 2.2.5 Proactive personality and meaning of work

Individuals with a proactive personality can work hard to control and manipulate the environment to pursue new information and practices to improve performance [16]. The employee will make active efforts not to give up in the face of challenges by taking the initiative. Employees with this proactive personality also always try to take jobs and position themselves in organizations where they can carry out tasks with a meaning of work high [23]. Such people will also take risks, if necessary, to find new jobs that align with their personality traits [38]. Until [20,23] states that individuals with a proactive personality will give a greater value to the meaning of work. [3] has also proven that a proactive personality has a significant effect on the meaning of work. Because some of these individuals already have openness to new experiences and jobs, they will be attracted to jobs and organizations that provide a high level of [3]. From the descriptions mentioned above, this study hypothesizes that:

H5: Proactive Personality has a significant effect on the Meaning of Work

#### 2.2.6 Proactive personality and work engagement

It is known that proactive personality has a positive correlation with work engagement as research [5] has suggested that proactive personality is related to work engagement in reciprocal relationships. According to [39] proactive personality must be positively related to work engagement because it will show individuals with a proactive personality involved in a work environment also tend to immerse themselves in their work. Furthermore, the actions of individuals with a proactive personality include always wanting to continue to learn about their work by identifying new ways to describe their strengths, and relentlessly reaching out to others such as co-workers, leaders, and customers, and it represents greater work engagement. It is also supported by the statement of [40] who stated that a proactive personality is a rather stable personality characteristic about showing initiative, persistence to bring about meaningful change and identifying opportunities and acting on them as anticipatory actions that employees take to influence themselves and/or their environment leading to work engagement. at workplace. From the descriptions mentioned above, this study hypothesizes that:

H6: Proactive Personality has a significant effect on Work Engagement

#### 2.2.7 Meaning of work and employee creativity

Research conducted by [29] has found that creativity increases along with acceptance and appreciation that their work has meaning. That way, it proves that there is a correlation between the meaning of work and employee creativity. Agreement between individual values and goals as well as organizational values and goals will increase the meaning of work, which makes employees more likely to display desired behaviors such as employee creativity [29]. Besides, when the meaning of work is high, then it can increase the contribution of employees to the organization by increasing other intrinsic motivations. Employees will likely find it difficult to develop creative ideas because their cognitive resources are insufficient to develop creative skills for their work area [38]. According to [3] meaning of work can become the target of an individual because it can support and encourage them to achieve integrated integrity which also leads to employee creativity. However, different subjects of the study will certainly produce different detailed findings [3]. From the descriptions mentioned above, this study hypothesizes that:

H7: Proactive Personality has a significant effect on Employee Creativity

#### 2.2.8 Work engagement and employee creativity

Employees with work engagement more often experience positive emotions that expand their intellectual and psychological resources which encourage them to explore new unconventional ways of doing their jobs [26]. This positive psychological experience can motivate employees to learn, assimilate new information and produce ingenious solutions and ideas and subsequently make them have higher energy needed to pursue creativity than those who are not involved [26]. To support this argument, [41] shows a significant relationship between work engagement and creativity. This is also supported by [42] who has researched the relationship between work engagement and creativity. According to the study, work-engaged employees often experience positive emotions that expand their momentary thought-action repertoire processes and generate personal resources, and those positive emotions facilitate creative behavior by cultivating a thirst to explore and assimilate new information [42]. From the descriptions mentioned above, this study hypothesizes that:

H8: Work Engagement has a significant effect on Employee Creativity

#### 2.2.9 Meaning of work mediating perceived organizational support and employee creativity

According to [43] intrinsic motivation tends to have a direct impact on employee creativity, meanwhile, extrinsic motivation will only increase if employees perceive their work to have meaning. This is following the statement [44] that " meaningfulness is regarded as an important component of intrinsic motivation " which means that meaningfulness is an important component of intrinsic motivation. Therefore, when employees perceive that their work has meaning, their intrinsic motivation and creativity also increase continuously [3]. When employees feel the meaning of work, they will consider it an important component of intrinsic motivation which also has the aim of utilizing the abilities, talents, and creativity of employees. As long as the organization delegates authority that affects employees to take advantage of these opportunities, they will have perceived organizational support that makes them feel that they must always try harder to cooperate with management. Supported again by the meaning of work, able to increase the desire of employees to be creatively involved. So that employees will be encouraged to have a desire to try harder to be creatively involved. Employees who feel limited and do not get organizational support tend to find it difficult to find the value of their work as a result there may be decreasing in the potential for creativity and innovation [45]. Furthermore, [3] in his research which also observed the relationship between perceived organizational support and employee creativity through the meaning of work as a mediator found that organizational support contributed to an increase in work meaningfulness which then had an impact on increasing employee creativity. Therefore, in this study, further observations will be made of how perceived organizational support affects employee creativity with the meaning of work as a mediator. From the descriptions mentioned above, this study hypothesizes that:

H9: Meaning of Work mediates the relationship between Perceived Organizational Support and Employee Creativity

#### 2.2.10 Work engagement mediating perceived organizational support and employee creativity

The sense of belonging to the organization causes employees to evaluate their work by considering the interests of the organization which makes employees prefer to link their interests with the organization as a common interest to develop trust in increasing their creativity for the organization [46]. According to [47] employees who experience perceived organizational support will interpret it twice as much as an organization that shows value and investment in its employees, and the organization’s concern for their welfare. Furthermore, employees will know their position as well as job content, and they will know their shortcomings well enough to create many good ideas for improvement that arise if their creativity is motivated [48]. Higher work engagement is observed to be positive when employees strongly agree with the organization, based on social exchange theory they will generate motivation to pay for their organization to be better as one of them refers to creativity [49]. [50] has shown that work engagement acts as a strategic driver to increase employee creativity, which is also able to be a driver in motivating employees to be able to focus their intelligence and increase their creativity. So from the findings of the experts above, it can be seen that organizational support has a significant impact on work engagement and the results then increase employee creativity. However, it is known that the mediating role of work engagement has not been supported by strong empirical evidence due to the lack of research. From the descriptions mentioned above, this study hypothesizes that:

H10: Work Engagement mediates the relationship of Perceived Organizational Support to Employee Creativity

#### 2.2.11. Meaning of work mediating proactive personality and employee creativity

Organizations mostly demand enthusiastic and proactive employees. Employees with a proactive personality are known to be more enthusiastic about improving their work performance through their efforts, and they are more determined to identify opportunities to achieve their goals without feeling constrained by internal or external constraints [51]. Employees who find their work meaningful and important will believe that they can feel creative, especially individuals who already have a determination to have a proactive personality [3]. Meaning of work is known to depend on the suitability of the diversity of the job to the personality of the employee [52]. Therefore, it is possible that employees who have a proactive personality also have high creativity. This happens because individuals with a proactive personality are better at managing their work roles with expectations, feeling the compatibility between their expertise and work values with the tasks they do in their daily work [53]. In addition, research by [29] found a correlation between proactive personality and employee creativity moderated by the meaning of work. In this study, it was stated that a proactive personality has an indirect impact on employee creativity through the meaning of work. From the descriptions mentioned above, this study hypothesizes that:

H11: Meaning of Work mediate the relationship between Proactive Personality and Employee Creativity

#### 2.2.12 Work engagement mediating proactive personality and employee creativity

Highly engaged employees often feel enthusiasm, excitement, and interest in their work, which then magnifies their thinking and actions [50]. Moreover, enrich their resources by expanding their suggestions and actions [54]. According to [16] being proactive means foreseeing things, stopping problems, and seizing opportunities by initiating efforts to stimulate and bring about change in work and having a different vision for the future such as boosting creativity. The positive emotions and cognitive states of work engagement are also beneficial for proactive employees because they can focus intelligence on their opportunities to increase creativity. A proactive personality has an impact on increasing the tendency of individuals to have high concern and enthusiasm for work. Meanwhile, creativity is related to innovative work behavior and individual capacity to create ideas. Then, work engagement is understood as the relationship between employees and the work in their organization which can later lead to work behavior that is full of enthusiasm and high effort. In short, a proactive personality increases employee engagement, and then the impact affects employee creativity. [50] has shown that work engagement can mediate organizational needs in influencing employee creativity. From the descriptions mentioned above, this study hypothesizes that:

H12: Work Engagement mediates the relationship between Proactive Personality and Employee Creativity

**Fig 1:**
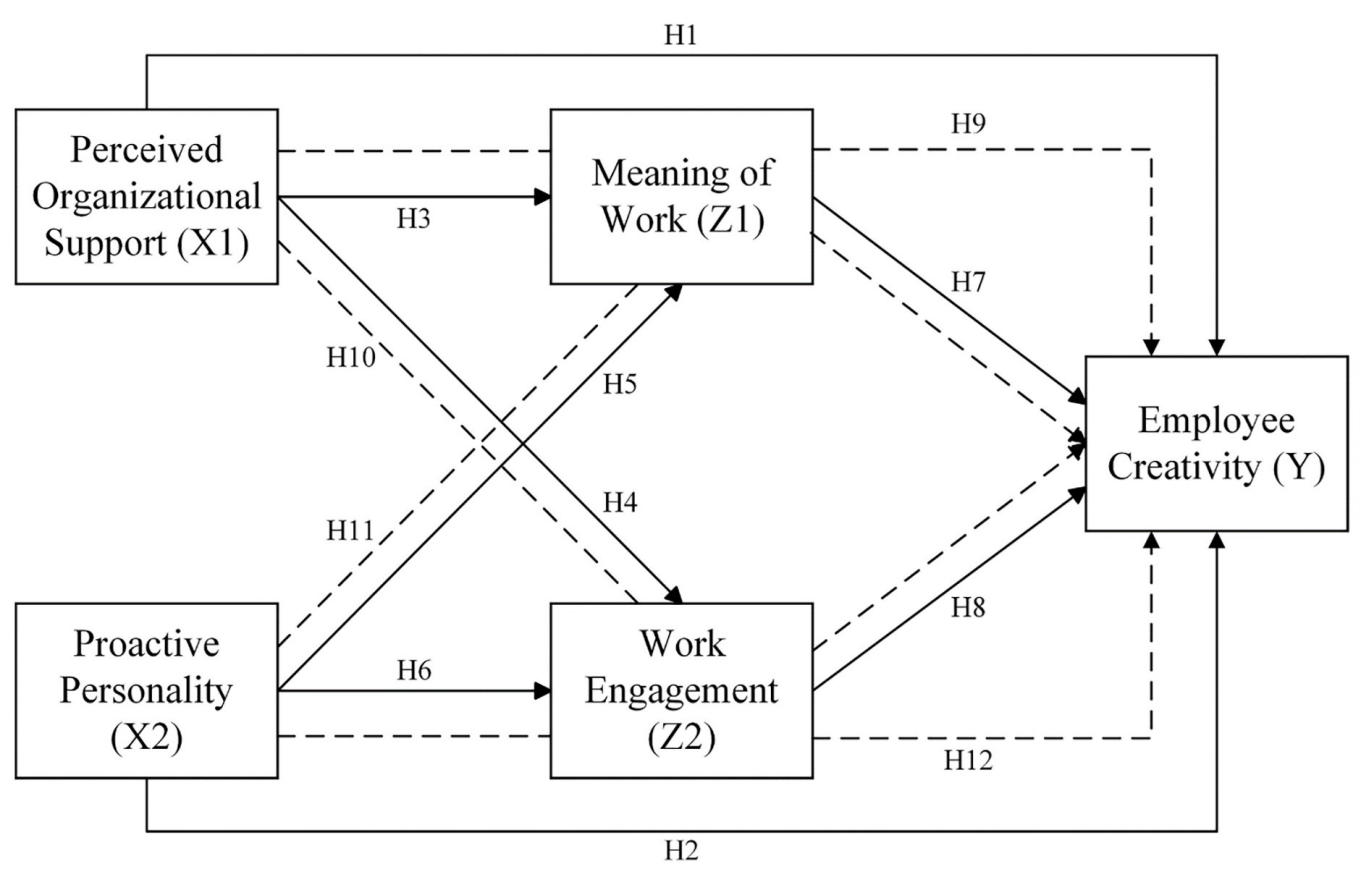
Conceptual framework.

The hypotheses were then conceptualized in the following framework (Fig 1).

## 3 Research methods

### 3.1 Research approach

This research was a quantitative explanatory study since variables were examined using hypotheses and path analysis models were employed. An explanatory research model is used to test a theory by collecting evidence that either supports or refutes it. This study’s population consisted of field reporters in the DKI Jakarta region of Indonesia. The sample of this study was 119 news reporters who were selected through a purposive sampling technique based on the following criteria, (a) members of the Alliance of Independent Journalists (AJI) and the Indonesian Television Journalists Association (IJTI); (b) reporters from 14 national private television stations; (c) a permanent news reporter; and (d) more than two years tenure. The journalists who were members of AJI and IJTI were selected because AJI and IJTI are professional organizations that oversee the television journalist profession hence the members were considered credible.

Furthermore, the data collection process was carried out by distributing questionnaires using Google Form media. The reason for choosing news reporters who worked for private television stations was because private television stations are organizations that rely on advertising as their main income. In contrast, state television stations receive a budget from the state and have a tendency to broadcast what is mandated by the state. Thus, observing creativity on state-owned stations is not appropriate.

As this is a non-interventional study, Universitas Airlangga’s Development and Innovation Institute of Publishing Journal and Intellectual Property Rights (LIPJIPHKI) determined that no ethical approval was needed. This institute is accountable for conducting research and guiding the development of new research products for the benefit of the community. This Institute has the power to provide ethical approval for Universitas Airlangga academics’ research. Following the organization’s policy, the chief executive, who represented the organization as a whole, also provided written informed consent. The consent was also validated by the Development and Innovation Institute for Publishing Journal and Intellectual Property Rights at Universitas Airlangga (LIPJIPHKI). However, the participants were also informed that their information would be kept strictly confidential and used for research purposes only.

### 3.2 Measurement

The independent variables in this study are Perceived Organizational Support (POS), and Proactive Personality (PP), then the dependent variable in this study is Employee Creativity (EC), and the mediator variables used in this study are Work Engagement (WE) and Meaning of Work (MOW). Perceived organizational support was measured with 8-item, one sample item was “The organization is very concerned with the welfare of employees” [3]. Furthermore, Proactive personality was measured by 10 items, one of them was “I saw opportunity earlier than anyone else” [18]. Meaning of work was measured with 15 items, such as "Work is seen as giving meaning to the individual" which represents importance of work, "Individual understands the job well" which represents understanding of work, "Individual understands the direction and purpose of work" which represents direction of work, and “Individuals often do not understand the function of work ®” which represents the purpose of work [21]. Work engagement was measured with 17 items, such as "Individuals feel empowered and have power towards their work" representing vigor, "Individuals have pride in their work" representing dedication, and "Individuals have a high focus at work" representing absorption [24]. Then, Employee Creativity was measured with 9 items, such as “Individuals have a desire to try new things” [32]. All of the questionnaire items mentioned above had been tested for reliability in previous studies and had a Cronbach alpha value above 0.60.

### 3.3 Data analysis technique

This study used Partial Least Squares-Structural Equation Modelling (PLS-SEM) as an analytical technique to determine the overall interaction between variables and emphasizes hypothesis testing in this study. PLS was used because it is a sophisticated analytical method that can be applied to various sizes of data, meaning the required sample size does not have to be large. This is in accordance with the number of samples in this study which amounted to 119 which was a relatively small number. In addition, no previous research had evaluated this research model in the setting of news reporters, hence the theory was still weak/had not been extensively tested. In PLS-SEM, there are two stages that must be completed: assessing the measurement model or external model by evaluating the validity and reliability of the structure of each indicator, and testing the hypothesis with the bootstrap resampling method and test statistics.

### 3.4 Data analysis

The respondents in this study were 119 field news reporters from a total of 14 private television stations in Indonesia. The characteristics used included age, gender, agency, last education, and marital status.

Based on Table 1, the majority of respondents were male (58%) and aged between 30–40 years old (50.4%). The last degree that they possessed were bachelor’s degree (84%), and most of them were married (65.5%). In this study, to use PLS-SEM for data analysis, it is necessary to go through an outer model evaluation and an inner model evaluation.

**Table 1:** Characteristics of respondents.

Characteristics	Classification	Amount	Percentage
Age	20–30 years old	49 people	41.2%
	30–40 years old	60 people	50.4%
	>40 years old	10 people	8.4%
Gender	Man	69 people	58.0%
	Woman	50 people	42.0%
Last education	Diploma	19 people	16.0%
	Bachelor	100 people	84.0%
Marital status	Married	78 people	65.5%
	Widower widow	1 person	0.8%
	Not married yet	40 people	33.6%

### Outer model evaluation

**Fig 2:**
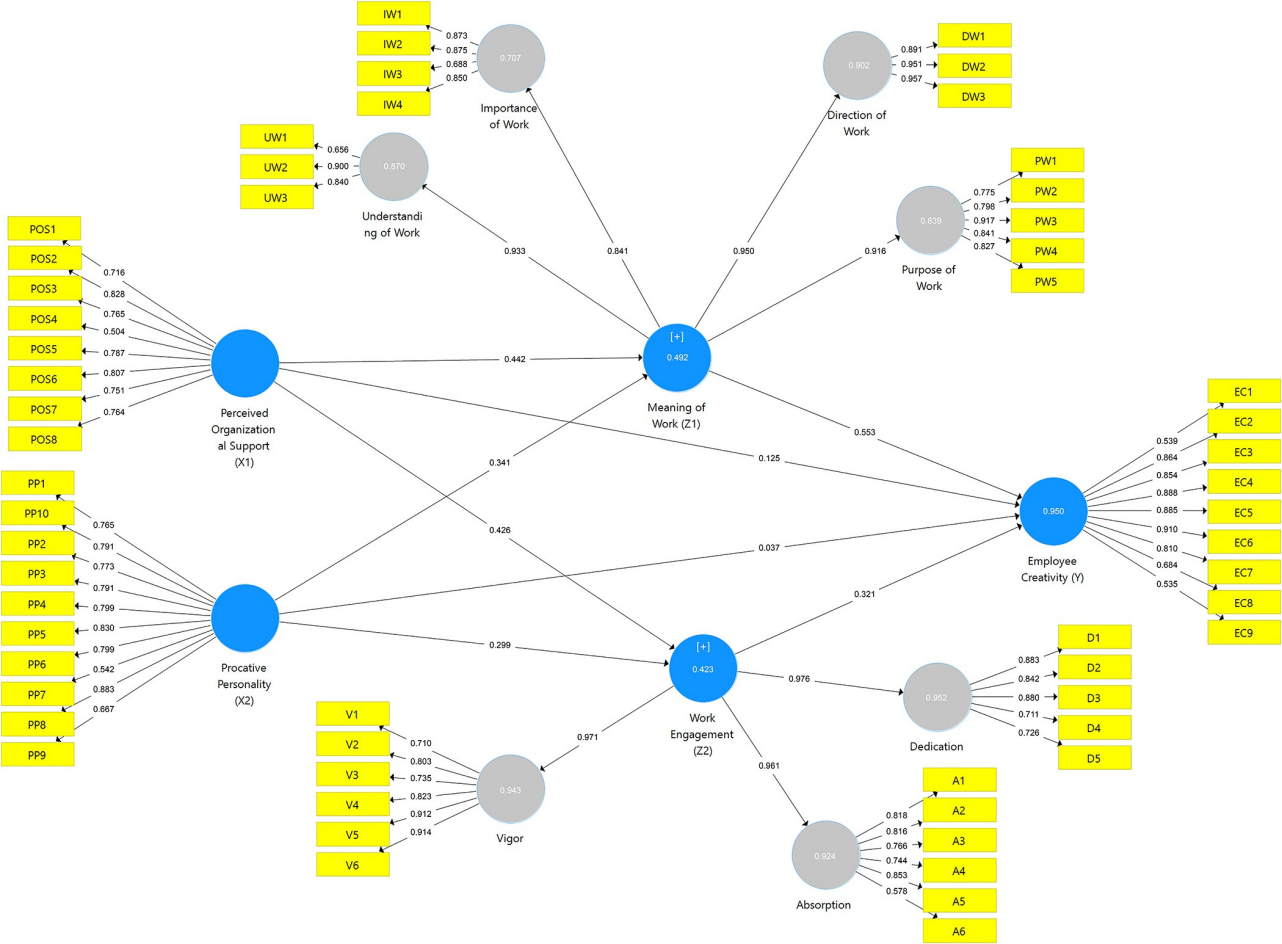
PLS algorithm. Estimation results.

The outer model evaluation goes through an evaluation of convergent validity, and composite reliability. In looking at the evaluation at the convergent validity stage, outer loading has a function to describe how big the relationship between indicators is with each construct. Based on Fig 2, all indicators on research variables already had an outer loading value > 0.50 [55]. Furthermore, the results of the convergent validity and internal consistency tests are shown in Table 2. Therefore, all indicators were valid in measuring perceived organizational support, proactive personality, the meaning of work, work engagement, and employee creativity variables and meet convergent validity to be used for further analysis.

**Table 2:** Outer model test.

Construct	Indicator		Validity Test			Reliability Test	
			*Outer Loading*	*AVE*	Note	Composite Reliability	Result
***Perceived Organizational Support* (X1)**	POS1		0.716	0.557	Valid	0.908	Reliable
	POS2		0.828		Valid		
	POS3		0.765		Valid		
	POS4		0.504		Valid		
	POS5		0.787		Valid		
	POS6		0.807		Valid		
	POS7		0.751		Valid		
	POS8		0.764		Valid		
***Proactive Personality* (X2)**	PP1		0.765	0.593	Valid	0.935	Reliable
	PP2		0.773		Valid		
	PP3		0.791		Valid		
	PP4		0.799		Valid		
	PP5		0.830		Valid		
	PP6		0.799		Valid		
	PP7		0.542		Valid		
	PP8		0.883		Valid		
	PP9		0.677		Valid		
	PP10		0.791		Valid		
***Meaning of Work* (Z1)**	*IW*	IW1	0.873	0.681	Valid	0.955	Reliable
		IW2	0.875		Valid		
		IW3	0.688		Valid		
		IW4	0.850		Valid		
	*UW*	UW1	0.656	0.649	Valid		
		UW2	0.900		Valid		
		UW3	0.840		Valid		
	*DW*	DW1	0.891	0.871	Valid		
		DW2	0.951		Valid		
		DW3	0.957		Valid		
	*PW*	PW1	0.775	0.694	Valid		
		PW2	0.798		Valid		
		PW3	0.917		Valid		
		PW4	0.841		Valid		
		PW5	0.827		Valid		
***Work Engagement* (Z2)**	*V*	V1	0.710	0.672	Valid	0.962	Reliable
		V2	0.803		Valid		
		V3	0.735		Valid		
		V4	0.823		Valid		
		V5	0.912		Valid		
		V6	0.914		Valid		
	*D*	D1	0.883	0.659	Valid		
		D2	0.842		Valid		
		D3	0.880		Valid		
		D4	0.711		Valid		
		D5	0.726		Valid		
	*A*	A1	0.818	0.589	Valid		
		A2	0.816		Valid		
		A3	0.766		Valid		
		A4	0.744		Valid		
		A5	0.853		Valid		
		A6	0.578		Valid		
***Employee Creativity* (Y)**	EC1		0.539	0.62	Valid	0.934	Reliable
	EC2		0.864		Valid		
	EC3		0.854		Valid		
	EC4		0.888		Valid		
	EC5		0.885		Valid		
	EC6		0.910		Valid		
	EC7		0.810		Valid		
	EC8		0.684		Valid		
	EC9		0.535		Valid		

After that, the discriminant validity test was carried out by comparing the value of the cross-loading item measuring the variable itself with other variables. The item is said to be valid if the cross-loading value is smaller in other variables. The results of the cross-loading calculation are presented in Table 3 below. It was known that all indicator items met discriminant validity because they had the largest cross loading value for the variables they form (bold values) and smaller in other variables. Thus, all indicators on perceived organizational support, proactive personality, meaning of work, work engagement, and employee creativity variables met discriminant validity.

**Table 3:** Cross loading test.

Construct		Indicator	X1	X2	Z1				Z2			Y
			*POS*	*PP*	*IW*	*UW*	*DW*	*PW*	*V*	*D*	*A*	*EC*
***Perceived Organizational Support* (X1)**		POS1	0.716	0.540	0.348	0.480	0.438	0.450	0.393	0.419	0.444	0.480
		POS2	0.828	0.556	0.438	0.549	0.501	0.436	0.500	0.442	0.414	0.573
		POS3	0.765	0.400	0.408	0.501	0.429	0.449	0.397	0.430	0.405	0.517
		POS4	0.504	0.279	0.260	0.320	0.274	0.212	0.316	0.320	0.269	0.320
		POS5	0.787	0.513	0.534	0.618	0.553	0.515	0.638	0.567	0.565	0.684
		POS6	0.807	0.449	0.431	0.540	0.523	0.475	0.458	0.494	0.457	0.556
		POS7	0.751	0.431	0.341	0.519	0.401	0.401	0.441	0.396	0.397	0.516
		POS8	0.764	0.319	0.295	0.399	0.354	0.393	0.330	0.361	0.355	0.407
***Proactive Personality* (X2)**		PP1	0.423	0.765	0.483	0.487	0.474	0.408	0.450	0.496	0.445	0.526
		PP2	0.358	0.773	0.358	0.456	0.391	0.331	0.410	0.356	0.378	0.435
		PP3	0.491	0.791	0.407	0.518	0.439	0.415	0.410	0.409	0.382	0.485
		PP4	0.416	0.799	0.459	0.493	0.466	0.393	0.470	0.451	0.435	0.501
		PP5	0.410	0.830	0.478	0.448	0.451	0.420	0.432	0.480	0.436	0.477
		PP6	0.388	0.799	0.310	0.386	0.391	0.359	0.352	0.360	0.334	0.407
		PP7	0.333	0.542	0.209	0.301	0.233	0.181	0.171	0.188	0.147	0.272
		PP8	0.530	0.883	0.463	0.519	0.522	0.441	0.459	0.490	0.438	0.547
		PP9	0.607	0.677	0.329	0.471	0.463	0.449	0.401	0.433	0.435	0.483
		PP10	0.582	0.791	0.457	0.549	0.554	0.399	0.503	0.462	0.414	0.569
***Meaning of Work* (Z1)**	*IW*	IW1	0.484	0.445	0.873	0.627	0.587	0.531	0.692	0.711	0.587	0.653
		IW2	0.454	0.451	0.875	0.623	0.579	0.512	0.710	0.668	0.624	0.638
		IW3	0.253	0.315	0.688	0.512	0.546	0.479	0.536	0.546	0.505	0.556
		IW4	0.519	0.508	0.850	0.662	0.694	0.568	0.669	0.718	0.635	0.709
	*UW*	UW1	0.587	0.492	0.500	0.656	0.483	0.456	0.431	0.469	0.433	0.528
		UW2	0.606	0.551	0.630	0.900	0.759	0.767	0.823	0.767	0.743	0.864
		UW3	0.464	0.448	0.645	0.840	0.870	0.676	0.912	0.843	0.807	0.854
	*DW*	DW1	0.562	0.450	0.729	0.836	0.891	0.741	0.914	0.903	0.823	0.888
		DW2	0.526	0.596	0.636	0.814	0.951	0.801	0.813	0.883	0.818	0.885
		DW3	0.582	0.584	0.685	0.855	0.957	0.795	0.832	0.882	0.817	0.910
	*PW*	PW1	0.481	0.417	0.633	0.767	0.733	0.775	0.717	0.745	0.677	0.810
		PW2	0.461	0.338	0.523	0.609	0.638	0.798	0.634	0.668	0.744	0.641
		PW3	0.509	0.446	0.531	0.695	0.740	0.917	0.691	0.746	0.853	0.739
		PW4	0.490	0.452	0.472	0.641	0.711	0.841	0.663	0.726	0.753	0.688
		PW5	0.432	0.442	0.475	0.611	0.643	0.827	0.610	0.660	0.818	0.649
***Work Engagement* (Z2)**	*V*	V1	0.454	0.451	0.875	0.623	0.579	0.512	0.710	0.668	0.624	0.638
		V2	0.406	0.343	0.486	0.686	0.726	0.559	0.803	0.696	0.684	0.713
		V3	0.445	0.429	0.563	0.642	0.622	0.657	0.735	0.650	0.656	0.671
		V4	0.606	0.551	0.630	0.900	0.759	0.767	0.823	0.767	0.743	0.864
		V5	0.464	0.448	0.645	0.840	0.870	0.676	0.912	0.843	0.807	0.854
		V6	0.562	0.450	0.729	0.836	0.891	0.741	0.914	0.903	0.823	0.888
	*D*	D1	0.413	0.412	0.621	0.718	0.807	0.662	0.783	0.883	0.814	0.773
		D2	0.425	0.360	0.633	0.731	0.790	0.620	0.772	0.842	0.723	0.772
		D3	0.572	0.565	0.699	0.845	0.937	0.805	0.840	0.880	0.821	0.910
		D4	0.484	0.445	0.873	0.627	0.587	0.531	0.692	0.711	0.587	0.653
		D5	0.490	0.452	0.472	0.641	0.711	0.841	0.663	0.726	0.753	0.688
	*A*	A1	0.432	0.442	0.475	0.611	0.643	0.827	0.610	0.660	0.818	0.649
		A2	0.438	0.385	0.600	0.780	0.816	0.665	0.857	0.801	0.816	0.805
		A3	0.379	0.390	0.508	0.624	0.690	0.590	0.680	0.768	0.766	0.670
		A4	0.461	0.338	0.523	0.609	0.638	0.798	0.634	0.668	0.744	0.641
		A5	0.509	0.446	0.531	0.695	0.740	0.917	0.691	0.746	0.853	0.739
		A6	0.396	0.366	0.695	0.529	0.461	0.418	0.572	0.534	0.578	0.506
*Employee Creativity* (Y)		EC1	0.664	0.488	0.423	0.505	0.431	0.403	0.450	0.418	0.417	0.539
		EC2	0.606	0.551	0.630	0.900	0.759	0.767	0.823	0.767	0.743	0.864
		EC3	0.464	0.448	0.645	0.840	0.870	0.676	0.912	0.843	0.807	0.854
		EC4	0.562	0.450	0.729	0.836	0.891	0.741	0.914	0.903	0.823	0.888
		EC5	0.526	0.596	0.636	0.814	0.951	0.801	0.813	0.883	0.818	0.885
		EC6	0.582	0.584	0.685	0.855	0.957	0.795	0.832	0.882	0.817	0.910
		EC7	0.481	0.417	0.633	0.767	0.733	0.775	0.717	0.745	0.677	0.810
		EC8	0.787	0.513	0.534	0.618	0.553	0.515	0.638	0.567	0.565	0.684
		EC9	0.360	0.389	0.576	0.459	0.431	0.405	0.455	0.476	0.403	0.535

The next evaluation in the outer model analysis was internal consistency. It tests the indicators consistency in measuring a construct. Internal consistency in PLS can use two measures, namely Cronbach’s alpha and composite reliability.

Table 4 shows that the internal consistency value of each research variable had a Cronbach’s Alpha value of more than 0.60 and a Composite Reliability value of more than 0.70. Thus, it could be concluded that each variable perceived organizational support, proactive personality, the meaning of work, work engagement, and employee creativity met good reliability.

**Table 4:** Internal consistency test.

Variable	Cronbach’s Alpha	Composite Reliability	Information
Perceived Organizational Support (X1)	0.883	0.908	Reliable
Proactive Personality (X2)	0.922	0.935	Reliable
Meaning of Work (Z1)	0.949	0.955	Reliable
Work Engagement (Z2)	0.957	0.962	Reliable
Employee Creativity (Y)	0.918	0.934	Reliable
Rule of thumb	0.60	0.70	

### Inner model evaluation

Inner model evaluation goes through an evaluation of the Coefficients of Determination (R2), Q-Square, and Path Coefficient assessment stages.

The adjusted R2 value for the meaning of work variable is 0.483, as shown in Table 5. This indicates that the influence of perceived organizational support and proactive personality on the meaning of work is 48.3%, which falls within the moderate category. Adjusted R-value 2 for the variable work engagement is 0.413, indicating that the influence of perceived organizational support and proactive personality on work engagement is 41.3% and belongs to the moderate range. Adjusted R-value2 for the employee creativity variable is 0.949%, which places perceived organizational support proactive personality, meaning of work, and work engagement in the large category (substantial).

**Table 5:** Coefficient of determination and Q-Square.

Endogenous Construct	R Square Adjusted	Q2 ^_^
Meaning of Work (Z1)	0.483	0.280
Work Engagement (Z2)	0.413	0.246
Employee Creativity (Y)	0.949	0.577

In addition, the measurements of Q2 were evaluated using a blindfold test, and a model is considered predictively relevant if the coefficient of Q2 is greater than 0. (56). Table 3 demonstrates that the Q2 values met the criterion of a value greater than zero, which qualified them as having a medium to large predictive relevance. The predictive relevance of the concept of employee creativity is high, indicating that the variables perceived organizational support, proactive personality, the meaning of work, and work engagement were strong predictors of a field news reporter’s employee creativity.

The hypothesis testing stage with path coefficient is the stage to determine whether or not there is a direct relationship between exogenous variables and endogenous variables used in research.

**Fig 3:**
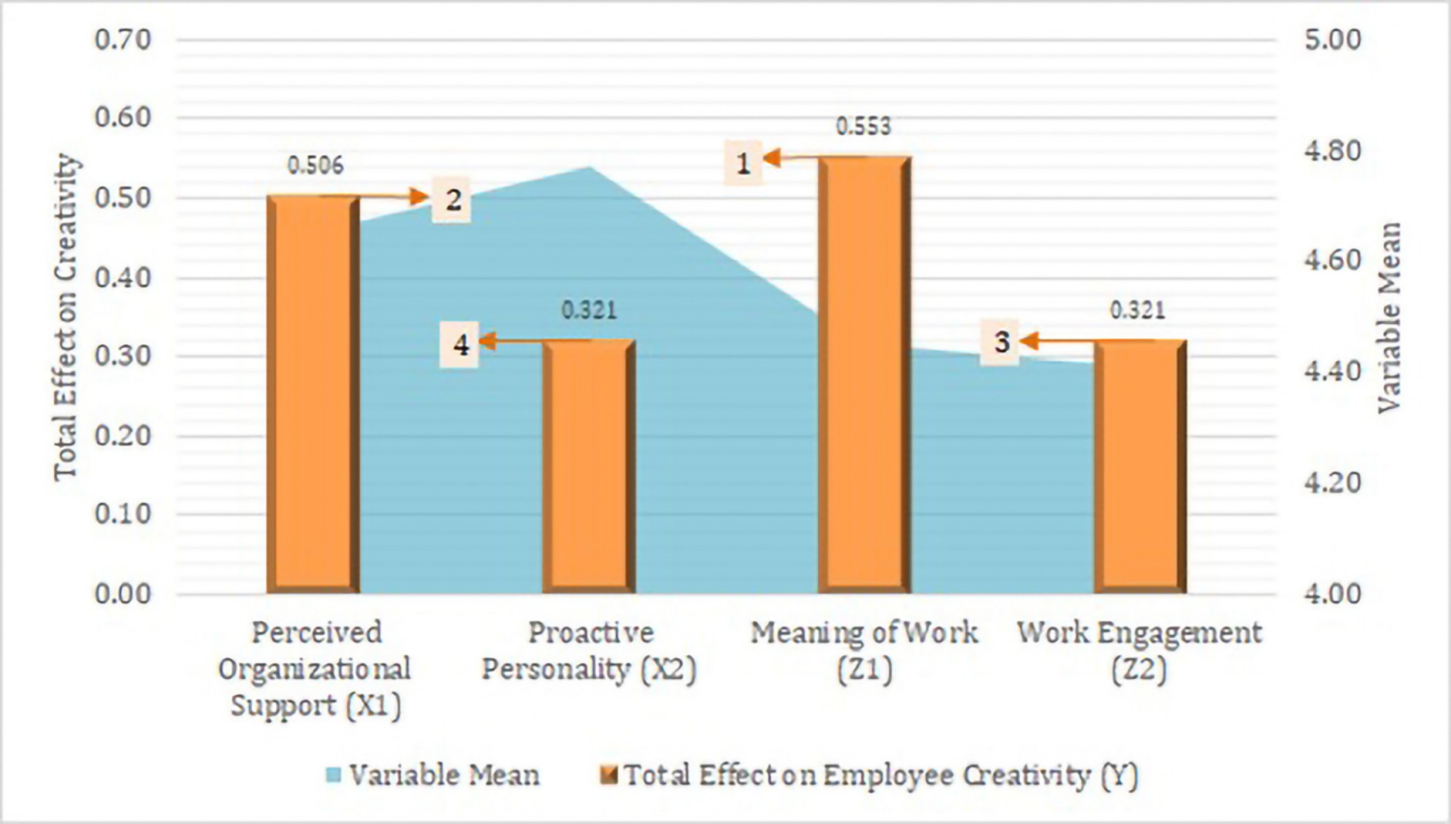
Total effect on employee creativity.

Based on Table 6, it is known that in the 2-tailed test, the research hypothesis can be accepted if the t-count (T-statistic) 1.96 or p-value is smaller than the error rate (α) 5%. Thus, there were eleven accepted hypotheses and one rejected hypothesis. Furthermore, all exogenous variables, both perceived organizational support and proactive personality, were significantly mediated by the meaning of work and work engagement. In this case, the meaning of work and work engagement fully mediated the influence of Proactive Personality on Employee Creativity. Meanwhile, the two mediating variables partially mediated the effect of Perceived Organizational Support on Employee Creativity (Fig 3).

**Table 6:** Path analysis of direct and indirect effects.

Path	Coefficient	T Statistics	P-value	Result
*Perceived Organizational Support* → *Employee Creativity*	0.125	3.812	0.000	Significant
*Proactive Personality* → *Employee Creativity*	0.037	1.337	0.182	Not Significant
*Perceived Organizational Support* → *Meaning Of Work*	0.442	6.718	0.000	Significant
*Perceived Organizational Support* → *Work Engagement*	0.426	5,998	0.000	Significant
*Proactive Personality* → *Meaning Of Work*	0.341	5.769	0.000	Significant
*Proactive Personality* → *Work Engagement*	0.299	4.860	0.000	Significant
*Meaning Of Work* → *Employee Creativity*	0.553	5.752	0.000	Significant
*Work Engagement* → *Employee Creativity*	0.321	3,431	0.001	Significant
*Perceived Organizational Support* → *Meaning Of Work* → *Employee Creativity*	0.244	-	0.000	Significant
*Perceived Organizational Support* → *Work Engagement* → *Employee Creativity*	0.137	-	0.000	Significant
*Proactive Personality* → *Meaning Of Work* → *Employee Creativity*	0.188	-	0.000	Significant
*Proactive Personality* → *Work Engagement* → *Employee Creativity*	0.096	-	0.000	Significant

X1 = Perceived Organizational Support, X2 = Proactive Personality, Z1 = Meaning of Work, Z2 = Work Engagement, Y = Employee Creativity.

The results of the comparative analysis of the mean and total effect variables concluded that to increase the creativity of field news reporters, the priorities from the highest to the lowest were meaning of work, perceived organizational support, work engagement, and lastly, proactive personality.

## 4 Discussion

Based on the results of statistical tests, it showed that perceived organizational support had a positive and significant effect on employee creativity with original sample = 0.125 (positive), T-statistics = 3.812 (≥1.96), p-value 0.000 (≤α = 5%). This was in line with research conducted by [33] which revealed that there was a positive correlation between perceived organizational support and employee creativity. It indicated that the greater the organizational support felt by the reporter, the more reporters’ creativity increased. Perceived organizational support could increase employee creativity behavior by increasing the reporters’ work interest, as well as in changing their intrinsic and extrinsic motivation. Reporters with perceived organizational support were encouraged to increase their sense of responsibility to achieve creativity by facing difficult goals, challenging tasks, and persisting when obstacles are encountered. The findings were in line with former research which proved that perceived organizational support had a significant impact on employee creativity working on various sectors [33]. Therefore, this study bolstered the empirical evidence of earlier studies indicating that perceived organizational support is relevant to employees’ creativity regardless of company sector.Subsequent research results show proactive personality no significant effect on employee creativity with original sample = 0.037 (positive), T-statistics = 1.337 (<1.96), p-value 0.182 (>α = 5%). This is not in line with the research of [16,17] who found that proactive personality was significantly related to employee creativity. The results of this study indicate that the higher the proactive personality of reporters, it does not have a real impact on increasing their creativity. One of the reasons is the existence of a code of ethics that regulates the work of news reporters. Reporters who work at private television stations in Indonesia are always required to be creative because their field of work relies heavily on creativity. Therefore, reporters will always create their creativity even if it is not in harmony with their proactive personality.

Then the results of the next statistical test showed that perceived organizational support had a positive and significant effect on the meaning of work with original sample = 0.442 (positive), T-statistics = 6.718 (≥1.96), p-value 0.000 (≤α = 5%). This is in line with the research by [29] which found a correlation between perceived organizational support and the meaning of work. The results of this study indicate that the greater the organizational support felt by the reporter, the higher the meaning of a job for the reporter. Perceived organizational support shows the perception of reporters working at private television stations in Indonesia related to the extent to which their place of work has recognized and appreciated their efforts and values, and has even cared about their well-being in an organization. Thus, it will trigger reporters to feel work becomes more meaningful. The results of the next study showed that perceived organizational support had a positive and significant effect on work engagement with the original sample = 0.426 (positive), T-statistics = 5.998 (≥1.96), p-value 0.000 (≤α = 5%). In line with research [23,37] perceived organizational support has a significant effect on work engagement. The results of this study indicate that the greater the organizational support felt by the reporter, the higher the reporter’s job involvement. The perceived positive value of the influence of perceived organizational support triggers reporters to increase the sense of obligation of employees to help the organization achieve its goals and to be able to contribute more to work performance through the resulting work engagement. Therefore, the findings in this study also support previous research which found a positive effect of perceived organizational support on work engagement in courier logistics companies [4,23,56]. Thus, the implications of perceived organizational support on work engagement prove relevant to be applied to a wider subject. In this case, previous studies have proven on the subject of logistics couriers and the results have been shown to be consistent with this study with the research subject of news reporters.

Furthermore, the results of the next statistical test showed that proactive personality had a positive and significant effect on the meaning of work with original sample = 0.341 (positive), T-statistics = 5.769 (≥1.96), p-value 0.000 (≤α = 5%). In line with the research of [20,23] which states that individuals with a proactive personality will give a greater value to the meaning of work. The results of this study indicate that the better the reporter’s proactive personality, the higher the meaning of a job for the reporter. Reporters with a proactive personality will always try to take a job and position themselves in the organization well which makes them feel a high meaning of work. Then the results of further research showed that proactive personality had a positive and significant effect on work engagement with original sample = 0.299 (positive), T-statistics = 4.860 (≥1.96), p-value 0.000 (≤α = 5%). In line with research [40] which states proactive personality significantly affects work engagement. The results of this study indicate that the better the reporter’s proactive personality, the higher the reporter’s work involvement. Proactive personality refers to showing initiative, and persistence to bring about meaningful change and identifying opportunities, and acting on them as anticipatory actions taken by reporters to influence themselves and/or their environment which can lead to work engagement in the workplace.

The results of the next statistical test showed that the meaning of work had a positive and significant effect on employee creativity with original sample = 0.553 (positive), T-statistics = 5.752 (≥1.96), p-value 0.000 (≤α = 5%). This is in line with the research of [3] which states that the meaning of work can support and encourage integrated integrity that leads to employee creativity. The results of this study indicate that the more reporters feel the meaning in their work, the higher the reporter creativity. When the reporters feel the meaning of work is high, they can increase other intrinsic motivation that can make it easier for them, or influence reporters to develop their creative ideas because they feel their work is meaningful. Then the results of further research showed that work engagement had a positive and significant effect on employee creativity with original sample = 0.321 (positive), T-statistics = 3.431 (≥1.96), p-value 0.000 (≤α = 5%). In line with the research of [42] which shows a significant relationship between work engagement and creativity. The results of this study indicate that the higher the reporter’s work involvement, the higher the creativity. The work engagement felt by the reporters made them experience positive emotions more often that could motivate them to learn, assimilate new information, and produce ingenious solutions and ideas to pursue creativity.

Furthermore, the results of this study on the relationship of indirect influence indicate that perceived organizational support is partially mediated by the meaning of work and work engagement. This shows that increasing the creativity of reporters can only increase perceived organizational support, but if the meaning of work and work engagement is also increased, the reporter’s creativity will increase even higher. Because perceived organizational support can have a direct impact on increasing reporter creativity, or indirectly through mediator variables. The results of this study indicate that organizational support contributes to improving the meaning of work and work engagement of reporters well. so that reporters are encouraged to have an impact on increasing employee creativity.

Furthermore, a proactive personality is fully mediation by the meaning of work and work engagement. This finding is different from some previous studies stated that proactive personality significantly affected employee creativity [3,57]. Proactive personality cannot affect employee creativity in news reporters, it can be caused by several factors. First is the existence of a journalistic code of ethics that limits and regulates the work of a news reporter. Even though a news reporter has a strong proactive personality, it does not mean he can change or exceed the provisions set out in the journalistic code of ethics. Then secondly, there are policies and business orientations of television stations that will determine what kind of broadcast will be broadcast to their viewers. This shows that increasing reporter creativity cannot only rely on a proactive personality but must also be supported by the meaning of work and work engagement so that reporter creativity can increase. Because efforts to increase creativity without paying attention to the meaning of work and work engagement the effect is not optimal in creating creativity. This can happen because the meaning of work can make reporters believe that they can feel creative, and when they feel work engagement, the reporters will focus their intelligence on their opportunities to increase creativity.

## 5 Conclusions and implications

### 5.1 Conclusion

Based on the results and discussions that have been carried out in this study, the conclusions that can be drawn from a direct influence are that perceived organizational support has a positive and significant effect on employee creativity, meaning of work, and work engagement. These findings reflect that news reporters who get support from the television station where they work show a higher level of creativity. This is because news reporters feel justice and also feel needed, so that their performance is better and ultimately has an impact on creativity. Not only that, support from television stations on news reporters will create emotional and cognitive bonds of reporters on their work. Therefore, the level of work engagement on news reporters is high. Then, the meaning of work is related to the level of news reporters in interpreting their work as valuable and valuable [58]. Furthermore, the support from the television station where the news reporter works makes news reporters interpret their work as meaningful. This is because the support of television stations, especially psychologically, provides encouragement for news reporters to feel meaningful in their work

Then proactive personality has a positive and significant effect on the meaning of work and work engagement but not on employee creativity. This is possible because even though news reporters have a proactive personality and try to change their environment, there is a code of ethics and binding work rules. For example, news reporters must write news according to the vision and mission of the television station concerned. In other words, proactive personality and intention to bring about change in the environment cannot have a direct impact on the creativity of reporters because they are limited by the code of ethics and rules of television stations.

Furthermore, the meaning of work and work engagement has a positive and significant effect on employee creativity. Meanwhile, the indirect effect shows that all of the hypotheses are accepted, indicating that perceived organizational support and proactive personality are significantly mediated by the meaning of work and work engagement on employee creativity. This shows that if news reporters who get organizational support have attachments and consider their work as valuable, then this perception leads news reporters to be more creative in their work. Not only that, news reporters who have a proactive personality also allow them to be more engaged with work and feel the meaning of their work. In the end, this has an impact in the form of increasing the creativity of news reporters. Reporters work in the television industry which has a very important role in creativity and content creation. Therefore, we need a study that looks at the factors that influence the creativity of reporters in achieving organizational goals well. All the results of this study indicate that reporters have been able to create creativity well in doing their work and in achieving organizational goals with perceived organizational support, proactive personality, the meaning of work, and work engagement. All variables used contribute well and are in line to preserve and enhance creativity.

### 5.2 Managerial implications

The results of this study can be taken into consideration for employees and television companies in creating and increasing employee creativity in the workplace. It is hoped that organizations need to increase their concern with employee welfare because it is their assessment of organizational policies and procedures, then employees are also expected to be able to show their ability to see opportunities earlier than others as a form of being proactive in the workplace. In this way, employees also need to view their work as being able to give meaning to each individual from what has been provided by the organization. When employees feel that their work is important, they should feel good involvement with their work until they feel the desire to work starting when they wake up. The main thing is that the organization needs to create revolutionary ideas in a field, and employees should pay attention to all the factors that can be positive for themselves to be able to help the organization make this happen.

### 5.3 Theoretical implication

Based on the discussion that has been described, this research can also be used as a recommendation for further research to analyse the effect of perceived organizational support and proactive personality on employee creativity with the meaning of work and work engagement as intervening variables in other organizational contexts. In addition, further research can also explore other determinants that are thought to be able to strengthen employee creativity. This is useful in developing new ideas that are useful for creativity and the innovation process that is needed by the company in creating value and working conditions that are full of creativity.
